# 7-MEGA™ inhibits adipogenesis in 3T3-L1 adipocytes and suppresses obesity in high-fat-diet-induced obese C57BL/6 mice

**DOI:** 10.1186/s12944-024-02175-0

**Published:** 2024-06-22

**Authors:** Yeong-Seon Won, Seon-Gyeong Bak, Nisansala Chandimali, Eun Hyun Park, Hyung-Jin Lim, Hyuck Se Kwon, Sang-Ik Park, Seung Jae Lee

**Affiliations:** 1 Division of Research Management, Department of Bioresource Industrialization, Honam National Institute of Biological Resource, Mokpo, 58762 Republic of Korea; 2 Functional Biomaterial Research Center, Korea Research Institute of Bioscience and Biotechnology, 181 Ipsin-Gil, Jeongeup, 56212 Republic of Korea; 3 Department of Applied Biotechnology, University of Science and Technology (UST), Daejeon, 34113 Republic of Korea; 4 Department of Veterinary Pathology, College of Veterinary Medicine and BK21 FOUR Program, Chonnam National University, Gwangju, 61186 Republic of Korea; 5 Scripps Korea Antibody Institute, Chuncheon, 24341 Republic of Korea; 6 R&D Team, Food & Supplement Health Claims, Vitech, Jeonju, 55365 Republic of Korea

**Keywords:** Adipocytes, Insulin, Lipid metabolism, Obesity, Palmitoleic acid, PPARs

## Abstract

**Background:**

Overweight, often known as obesity, is the abnormal and excessive accumulation of fat that exposes the health of a person at risk by increasing the likelihood that they may experience many chronic conditions. Consequently, obesity has become a global health threat, presenting serious health issues, and attracting a lot of attention in the healthcare profession and the scientific community.

**Method:**

This study aims to explore the anti-adipogenic properties of 7-MEGA™ in an attempt to address obesity, using both in vitro and in vivo research. The effects of 7MEGA™ at three distinct concentrations were investigated in obese mice who were given a high-fat diet (HFD) and 3T3-L1 adipocytes.

**Results:**

7MEGA™ decreased the total fat mass, overall body weight, and the perirenal and subcutaneous white adipose tissue (PWAT and SWAT) contents in HFD mice. Additionally, 7MEGA™ showed promise in improving the metabolic health of individuals with obesity and regulate the levels of insulin hormone, pro-inflammatory cytokines and adipokines. Furthermore, Peroxisome proliferator-activated receptors (PPAR) α and γ, Uncoupling Protein 1 (UCP-1), Sterol Regulatory Element-Binding Protein 1 (SREBP-1), Fatty Acid-Binding Protein 4 (FABP4), Fatty Acid Synthase (FAS), Acetyl-CoA Carboxylase (ACC), Stearoyl-CoA Desaturase-1 (SCD-1) and CCAAT/Enhancer-Binding Protein (C/EBPα) were among the adipogenic regulators that 7MEGA™ could regulate.

**Conclusion:**

In summary, this study uncovered that 7MEGA™ demonstrates anti-adipogenic and anti-obesity effects, suggesting its potential in combating obesity.

**Supplementary Information:**

The online version contains supplementary material available at 10.1186/s12944-024-02175-0.

## Background

The incidence of obesity has been progressively rising worldwide in recent years, making it a serious global health concern. It is linked to a greater death rate when compared to underweight conditions and malnutrition [1, 2]. Adipose tissue, commonly known as fat tissue, is a key factor in the development and progression of obesity [3]. The hallmark of obesity is an excessive accumulation of adipose tissue, which raises the mass of body fat [4]. Adipose tissue can be classified as white adipose tissue (WAT) and brown adipose tissue (BAT) [5]. WAT consists mainly of adipocytes and can be further categorized into subcutaneous white adipose tissue (SWAT) and visceral white adipose tissue (VWAT). Adipocytes are formed through a process known as adipogenesis [6].

Adipocytes primarily absorb excess energy, when the energy intake is high, and release it when energy is insufficient [7]. When energy intake surpasses energy expenditure over a sustained period of time, excess calories are retained as triglycerides in adipose tissue, which causes obesity and weight increase [7, 8]. Additionally, adipocytes generate pro-inflammatory cytokines and adipokines such as Interferon Gamma (IFN-γ), Interleukin 1 Beta (IL-1β) and leptin, IL-6, Tumour Necrosis Factor (TNF) α and adiponectin that affect systemic metabolism [9, 10]. However, dysregulated adipogenesis contributes to the dysfunction of adipocytes thereby dysregulating the production and secretion of adipokines, which contribute to the pathophysiology of obesity by promoting insulin resistance, inflammation, and metabolic disturbances [9, 11]. Therefore, improving lipid metabolism, including the regulation of adipogenesis, is considered as one of the most common strategies for the treatment of obesity. Currently, efforts are being made to find compounds that can improve them [12]. In particular, the impacts of natural substances on lipid metabolism, adipogenesis, obesity, dyslipidaemia, diabetes and their molecular signalling mechanisms have been extensively studied [13, 14].

Recently, several studies have shown that orally administered palmitoleic acid (PA) improves insulin sensitivity and suppresses hepatosteatosis and inflammation in type II diabetic mice [15]. Moreover, several studies have demonstrated the multifaceted effects of PA in both 3T3-L1 adipocytes and obese mice. PA, an omega-7 fatty acid, exhibits beneficial metabolic effects including increased Glucose Transporter (GLUT) 4 content and glucose uptake, enhanced lipolysis and lipase activity via PPARα activation, and promotion of anti-hypertrophic and anti-inflammatory responses in adipose-derived stromal cells. Moreover, PA supplementation has been demonstrated to enhance fatty acid oxidation, oxygen consumption, and ATP production in white adipocytes. It also reduces hepatic steatosis and regulates both fatty acid oxidation and lipogenesis in the adipose tissue of obese mice [16–20]. Further, purified palmitoleic acid supplementation had a beneficial effect on improving serum lipoprotein profiles and systemic inflammation in adults with dyslipidaemia [14, 21]. Recent reports have suggested that omega-7 is a novel adipose-derived lipokine, which is an important regulator of lipogenesis, insulin action, and homeostasis [22, 23]. These findings collectively suggest the potential therapeutic implications of PA in combating obesity-associated metabolic dysregulation.

Furthermore, previous studies have investigated the effects of 7-MEGA™, a highly concentrated PA, as a whole compound on oxidative stress, inflammation, and skin regeneration [24–26]. However, there is a gap in the literature regarding its impact on adipogenesis or metabolism related to obesity. Therefore, the current study was designed to fill this gap by elucidating the potential anti-obesity mechanisms of 7-MEGA™ in both cellular and animal models. The findings of this research demonstrate that 7-MEGA™ exhibits promise as a therapeutic approach for obesity-related conditions by influencing adipocyte function. This includes inhibiting the formation of fat cells and reducing lipid accumulation. Therefore, results suggest that 7-MEGA has the potential to serve as a candidate ingredient for the development of functional foods.

## Materials and methods

### Preparation of 7-MEGA™

**Fig. 1:**
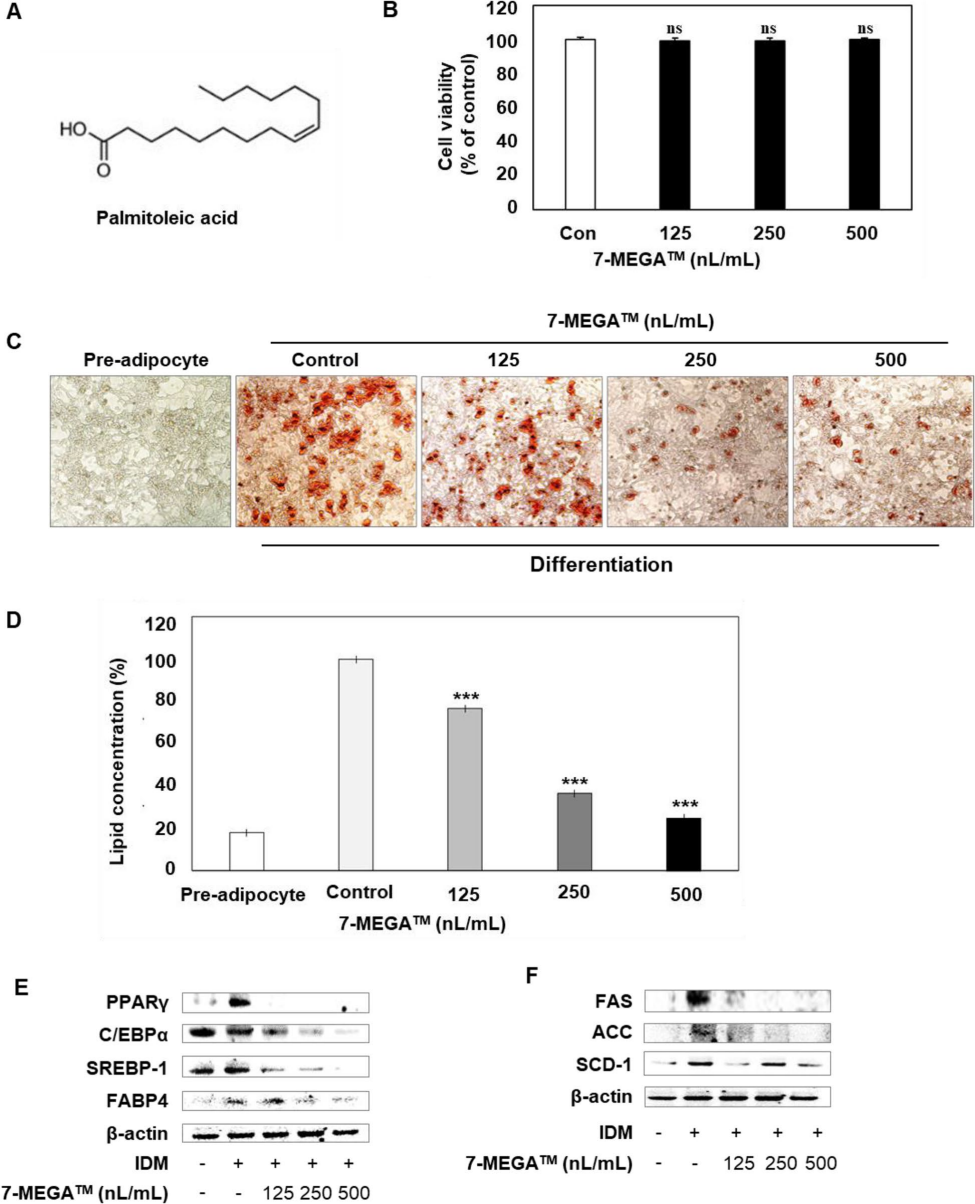
Effects of 7-MEGA™ on lipid accumulation of 3T3-L1 adipocytes and adipogenesis-related markers. **A** Chemical structure of palmitoleic acid. **B** Cell viability of 3T3-L1 adipocytes treated with increasing concentrations (125, 250 and 500nL/mL) of 7-MEGA™, as determined by the WST-1 assay. **C** Representative images of Oil Red O staining showing adipocyte differentiation after treatment with 125, 250 and 500nL/mL of 7-MEGA separately. **D** Quantification of lipid accumulation in adipocytes treated with different concentrations of 7-MEGA™ using the Oil Red O staining assay. **E**, **F** Western blot analysis of protein expression levels of adipogenesis-related markers, PPARγ, C/EBPα, SREBP-1, FABP4, FAS, ACC, and SCD-1 in 3T3-L1 adipocytes treated with increasing concentrations of 7-MEGA™. Densitometric quantification of protein expression levels normalized to loading control (β-actin). Data are presented as mean ± SD. *** *P* < 0.001 VS. the control group

AlaskOmega® Omega 7, also known as Omega‑7 fatty acid or 7‑MEGA™, is a highly concentrated palmitoleic acid (C16:1) [25]. 7-MEGA™ (purity 98.5% of omega-7, containing > 500 mg/g of palmitoleic acid (Fig. 1A, Supplementary Table 1), specific gravity- 0.894) was obtained from Wiley Companies in Eastern Ohio, USA. The raw material of 7-MEGA™, which is Alaska pollock, was recovered from the Alaskan Bering Sea, and was subsequently processed, purified, and concentrated to produce 7-MEGA™ [27]. Typical fatty acid profiles of 7‑MEGA™ are provided in supplementary Table 1. 7-MEGA™ was dissolved in 70% ethanol and stocked at − 80 °C. 125, 250, and 500 nL/mL of 7-MEGATM were used for the in vitro experiments, to enhance the precision and consistency of experimental procedures, thereby facilitating more effective extrapolation of results. In the animal experiment, 7-MEGA™ was administered at a volume of 10 mL/kg, in accordance with guidelines [28]. Prior to administration, each group’s defined concentration of 7-MEGA™ was dissolved in 1.5% Cyclodextrin solution (Samchun Chemical Co., Ltd., Seoul, Republic of Korea).

### Culture and differentiation of 3T3-L1 cells

3T3-L1 cells were purchased from American Type Culture Collection (ATCC, Rockville, MD, USA) and cultured in DMEM supplemented with 10% fetal calf serum (FCS), 10 mg/mL P/S (penicillin/streptomycin) in an incubator with a humidified atmosphere containing 5% CO_2_ set at 37 °C. Preadipocytes were plated at a concentration of 2.5 × 10^5^ cells/well in 6-well-plates. When the cell density appeared in 100% confluence, cells were replaced with differentiation DMEM medium containing 1 μg/mL insulin, 5 μM dexamethasone, and 0.5 mM 3-isobutyl-1-methylxanthine (IBMX) (IDM medium). After 3 days, cells were replaced with culture medium containing 10 μg/mL Insulin and the medium was changed every 2 days. During the induction of differentiation, cells were treated with 125, 250, and 500nL/mL of 7-MEGA™ on days 0–9.

### Oil-red O staining and microscopic analysis

Differentiated 3T3-L1 adipocytes on day 9 were fixed with 4% formalin solution (Bio-solution, Suwon, Republic of Korea) for 1 h. After washing with distilled water, cells were stained with 0.5% Oil Red O in isopropanol and incubated at room temperature for 1 h. They were then washed 4 times with distilled water. Stained mature adipocytes were assessed using a fluorescence microscope (Olympus Optical Co., Ltd., Tokyo, Japan). After visualization, 100% isopropyl alcohol was added to the plates, which were incubated for 10 min at room temperature. Absorption was measured in duplicate at 520 nm on a microplate reader (Molecular Devices, Sunnyvale, CA, USA).

### Assessment of cell viability using WST-1 assay

3T3-L1 cells were seeded at a concentration of 2.5 × 10^5^ cells/well in 96-well-plates and incubated for 24 h. Cells were then treated with 125, 250, and 500nL/mL 7-MEGA™ for 72 h. Next, 10 µL/well of cell proliferation reagent WST-1 (DoGenBio, Seoul, Repulic of Korea) was added in the plates and incubated for 4 h at 37 °C and 5% CO_2_. Absorbance was measured in duplicate at 460 nm on a microplate reader.

### Western blot analysis

Proteins from differentiated 3T3-L1 adipocytes on day 9 and PWAT in HFD mice, with or without 7-MEGA™ treatment, were harvested using lysis buffer (Cell Signalling Technology, Danvers, MA, USA), and the protein contents were analysed using a DC protein assay kit (Bio-Rad, Contra Costa County, CA, USA). Proteins were loaded onto a gel at a concentration of 30 μg of protein per lane and separated by 4–12% SDS-PAGE. After blocking with 5% bovine serum albumin (BSA) for 1 h at 37 °C, the membranes were incubated overnight with primary antibody at 4 °C. Finally, the membranes were incubated with secondary antibodies and developed using a West-Queen RTS Western Blot Detection Kit (iNtRON Bio., Seongnam, Republic of Korea).

### Animals and diets

All animal experiments were approved by the Institutional Animal Care and Use Committee of the Korea Food Research Institute (KRIBB-AEC-22266, KRIBB-AEC-23231). Female 8-weeks-old C57BL/6 mice were purchased from Orient Bio, Inc., (Seongnam, Republic of Korea). The mice were randomly divided into 6 groups (5 mice per group): Negative Control (NC) group without high-fat diets, High-fat-diet (HFD) group, HFD groups supplemented with 50 mg/kg, 100 mg/kg, and 150 mg/kg of 7-MEGA™ (7MEGA50, 7MEGA100, 7MEGA150), and HFD group supplemented with 200 mg/kg of *Garcinia Cambogia* (GC) as a positive control. The mice were housed in a laminar air flow room maintained at a temperature of 22 ± 2 °C and relative humidity of 55 ± 5%, with a 12 h light–dark cycle throughout the study. They were administered 50 mg/kg,100 mg/kg and 150 mg/kg of 7-MEGA™ or 200 mg/kg of GC orally 5 times a week for 8 weeks. Obesity groups were fed 60% HFD intake, and body weight was monitored once per week. At the end of the experimental period, mice were fasted overnight. Organs, including PWAT, SWAT, and liver, were collected and weighed.

### Dual-energy X-ray absorptiometry (DEXA)

The percentage of body mass as fat (%fat) in mice with or without HFD and 7-MEGA™ intake was assessed using a Dual-energy X-ray absorptiometry (DEXA, Medikors, Republic of Korea) according to the instructions of manufacture.

### Enzyme-linked immunosorbent assay (ELISA)

Serum analysis was conducted to assess systemic changes induced by the treatment. This analysis provided insights into the overall physiological response to 7-MEGA™ treatment, including potential alterations in circulating adipokine and cytokine levels, reflecting broader metabolic and inflammatory changes beyond the local effects observed in cell culture or ex vivo tissue models. Obesity-related biomarkers including insulin, adiponectin, leptin, IFN-γ, IL-1β, IL-6, and TNF-α were quantified in the serum using ELISA kits obtained from Morinaga Institute of Biological Science, Inc. (Yokohama, Japan); Crystal Chem, Inc. (IL, USA); Abcam (Cambridge, UK); and R&D Systems (MN, USA), following the manufacturers’ instructions. Absorbance was measured at 450 nm using a microplate reader.

### Statistical analysis

Data values are presented as the mean ± SD of measurements obtained in triplicate. Significant differences between experimental and control groups were determined by performing one-way ANOVA using Tukey’s multiple comparisons test and are reported as ** *P* < 0.01 and *** *P* < 0.001. The analyses were performed with Prism software (GraphPad, CA, USA).

## Results

### 7‑MEGA™ inhibits adipogenesis of 3T3-L1 adipocytes

First, the current study examined the impact of 7‑MEGA™ on the viability of 3T3-L1 preadipocytes. The cells were cultured and treated with 125, 250, and 500nL/mL 7‑MEGA™ for 72 h. Cell viability was determined using the WST-1 assay and 7‑MEGA™ showed no cytotoxicity at concentrations tested in 3T3-L1 adipocytes (Fig. 1B). Next, the 3T3-L1 cells were differentiated in IDM medium, either with or without 125, 250, or 500nL/mL 7‑MEGA™. Oil Red O staining was used to visualized and quantified the reduction in lipid accumulation due to 7‑MEGA™ in 3T3-L1 adipocytes. 7-MEGA™ markedly reduced lipid accumulation in 3T3-L1 adipocytes in a manner dependent on the dose (Fig. 1C, D). Therefore, these results demonstrate that 7‑MEGA™ reduces the accumulation of fat molecules in adipocytes and the differentiation of adipocytes.

### 7‑MEGA™ regulates the adipogenic transcriptional regulators and enzymes in 3T3-L1 adipocytes

Next, to investigate the impact of 7MEGA™ on adipogenic transcriptional regulators and enzymes western blot was conducted. We evaluated the PPARγ, C/EBPα, SREBP-1, FABP4, FAS, ACC, and SCD-1 expressions in 3T3-L1 adipocytes with or without 7MEGA™. The findings revealed that the addition of IDM medium increase all expression levels. However, after receiving 7-MEGA™ treatment, the protein levels of these regulators and enzymes were decreased. (Fig. 1E, F). These results indicate that 7‑MEGA™ regulates the adipogenesis by modulating adipogenic transcriptional regulators and enzymes during adipocyte differentiation.

### Body weight gain of HFD mice was reduced by 7MEGA™

**Fig. 2:**
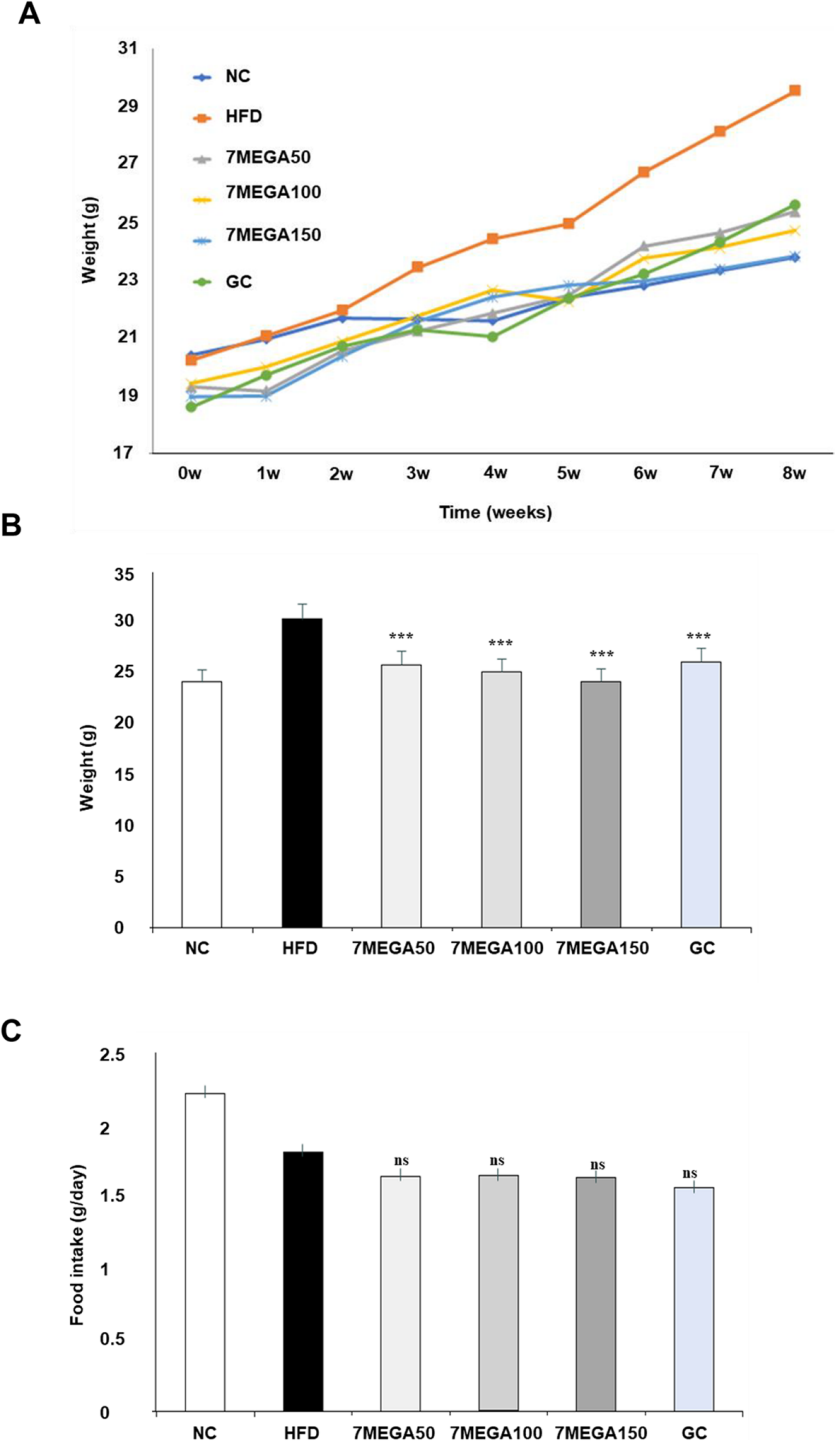
Effects of oral administration of 7-MEGA™ on body weight and food intake in HFD Mice. **A** Average body weights of mice in the Negative Control (NC) group, HFD group, and HFD groups orally administered with 50 mg/kg, 100 mg/kg, and 150 mg/kg of 7-MEGA™ daily for 8 weeks. **B** Quantified average weights of the aforementioned groups, showing the differences in body weight changes over the 8-week period. Body weights were measured weekly. **C** Graph illustrating the average food intake of mice in the NC, HFD, and HFD groups treated with varying doses of 7-MEGA™. Food intake was monitored weekly and is expressed as the mean consumption per mouse per day. Data are presented as mean ± SD. *** *P* < 0.001 VS. the control group

To expand the knowledge on the effects of 7MEGA™ in vivo experiments were conducted. First, weather 7MEGA™ regulates the body weight of obese individuals were examined. For that, HFD mice were administered 7‑MEGA™ doses of 50 (7MEGA50), 100 (7MEGA100) or 150 (7MEGA150) mg/kg/day orally for 8 weeks. A group fed a normal diet is used as the negative control (NC). The positive control was a group treated with 200 mg/kg of Garcinia Cambogia (GC). As shown in Fig. 2A & B, the body weight gain of HFD mice administered 7‑MEGA™ was reduced compared to HFD mice without 7‑MEGA™ administration. In addition, the food intakes of the HFD groups that received 7MEGA™ were marginally lower than those of the HFD group that did not receive 7-MEGA™ (Fig. 2C). These findings showed that 7-MEGA™ has a potential to reduce the body weight and food consumption of obese individuals.

### 7-MEGA™ attenuates fat mass accumulation in HFD mice

**Fig. 3:**
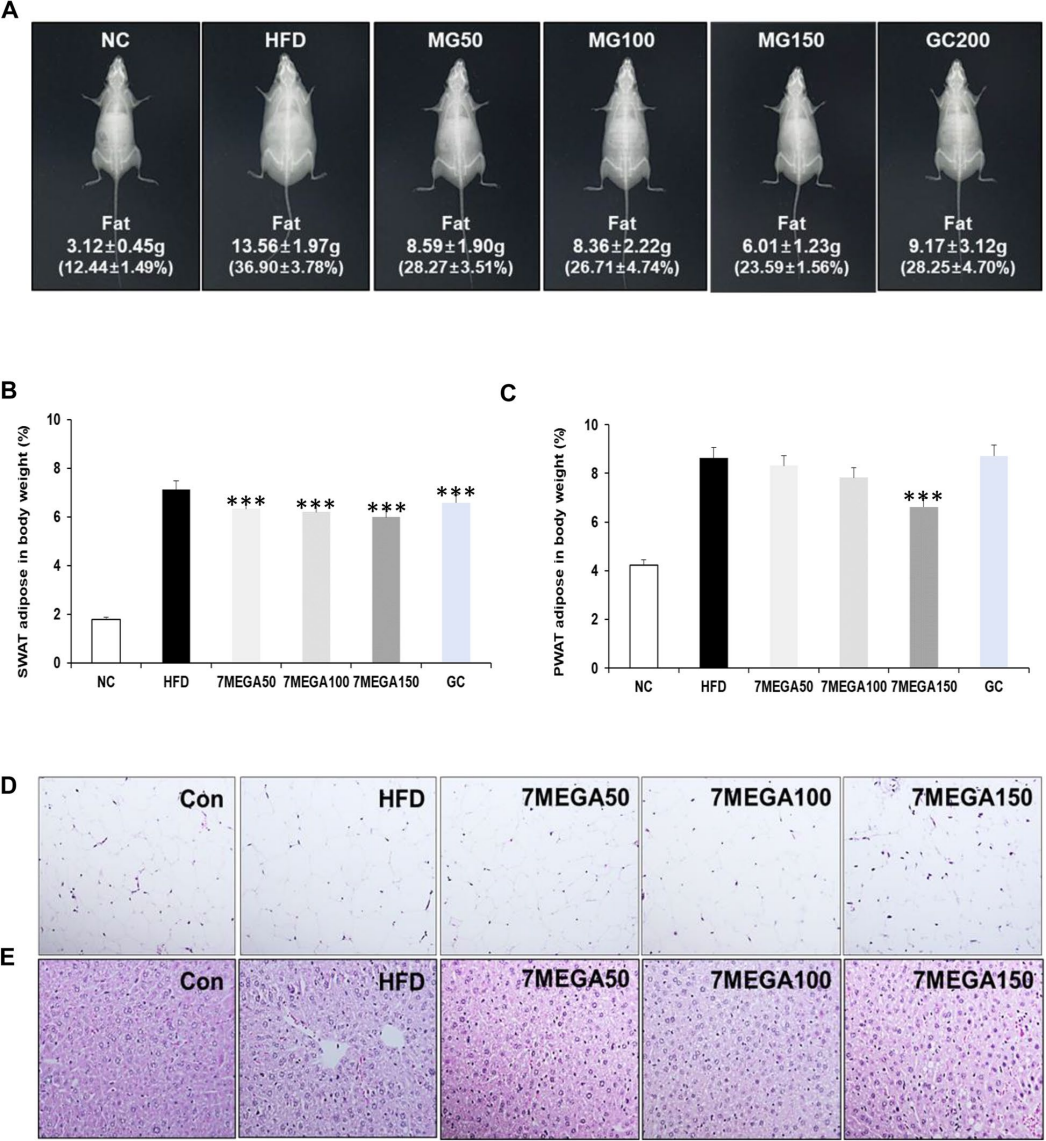
Effects of 7-MEGA™ on fat mass accumulation in HFD mice. **A** Fat mass accumulation of mice in the NC, HFD, and HFD groups administered with 7-MEGA™, measured by dual-energy X-ray absorptiometry (DEXA). Data are presented as mean values. **B** Average weights per body weight (g/g) of SWAT and **C** PWAT of mice in the NC, HFD, and HFD groups treated with 7-MEGA™. **D** Representative images of hematoxylin and eosin (H&E) staining of SWAT **E** and liver of mice. Data are presented as mean ± standard deviation (SD). *** *P* < 0.001 VS. the HFD group

Consuming more calories than expended can lead to the accumulation of excess calories in the body as fat, resulting in weight gain [29]. Therefore, whether the weight gain observed in HFD mice is attributable to fat accumulation and whether 7MEGA™ can reduce fat accumulation in the body was examined. First, dual x-ray absorptiometry (DEXA) was used to observe the accumulation of fat in mice. The HFD group had a higher fat weight than the NC group, as shown in Fig. 3A. However, the daily administration of 7-MEGA™ considerably decreased the accumulation of fat mass in HFD mice, surpassing even the positive control group (GC). Moreover, weights of both PWAT and SWAT exhibited significant increase in the HFD group compared to the NC group, however the weight decreased in the HFD groups which administered 7-MEGA™ in a manner dependent on the concentration (Fig. 3B & C).

As fat accumulates in the body, WAT undergoes a process of tissue remodelling characterized by an increase in the size of adipocytes, known as hypertrophy [30]. Thus, PWAT was examined to find out whether the adipocyte of HFD mice exhibited signs of hypertrophic conditions. The PWAT staining of HFD mice showed larger adipocytes, as shown in Fig. 3D, while mice administered 7MEGA™ showed adipocytes of normal size. This indicates that 7MEGA™ efficiently inhibits the accumulation of fat in PWAT. In order to see the accumulation of fat in the liver tissues, the liver tissues were further assessed. When liver tissues from the HFD group were stained with H&E, the accumulation of fat, white/pale colour regions, known as steatosis was visible [31]. Steatosis is a disorder that occurs when triglycerides accumulate in the tissues of the liver. However, steatosis disappeared in the groups that received oral 7-MEGA™ (Fig. 3E). All of these findings demonstrated that 7-MEGA™ reduces the fat mass accumulation in obese HFD mice.

### 7‑MEGA™ reduces the release of adipokine and pro-inflammatory cytokines by adipose tissues

**Fig. 4:**
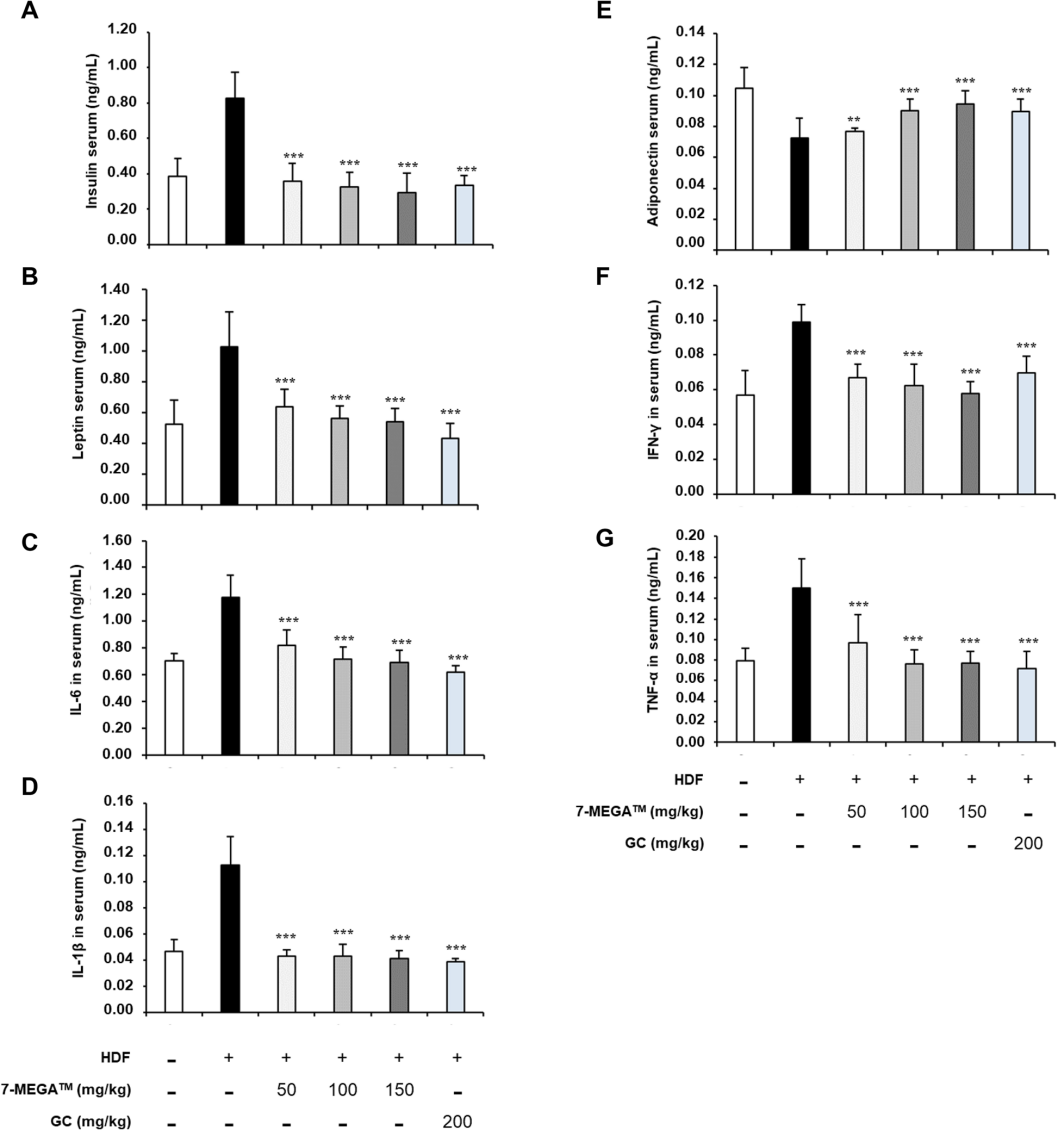
7-MEGA™ modulates the level of insulin, TNF‑α and adipokines. ELISA to quantitatively measure the levels of (**A**) Insulin (**B**) Adiponectin (**C**) Leptin (**D**) IFN‑γ (**E**) IL‑6 (**F**) TNF‑α and (**G**) IL‑1β in NC, HFD and HFD groups orally administered with 7-MEGA™ over an 8-week period. *** *P* < 0.001 VS. the HFD group

When fat mass increases, either adipocytes or macrophages infiltrating adipose tissue release adipokines and pro-inflammatory cytokines, which lead to low-grade chronic inflammation, insulin resistance, and the emergence of obesity-related disorders [10]. Therefore, ELISA was utilized to monitor the levels of key adipokines and pro-inflammatory cytokines, including leptin, IL-6, IL-1β, TNF-α, adiponectin, and IFN‑γ. Except for adiponectin, all other expression levels were increased in HFD mice, indicating fat mass accumulation. However, the administration of 7MEGA™ reduced the expression levels (Fig. 4B, C, D, F & G), demonstrating its ability to inhibit fat mass accumulation and thereby suppress the secretion of these factors. On the other hand, the findings showed that the serum of HFD mice contained lower amounts of adiponectin, which increased when 7-MEGA™ was given to them (Fig. 4E). Consistent with these findings, prior research has shown that mice deficient in adiponectin demonstrate elevated levels of fat accumulation. Therefore, these results prove that 7-MEGA™ can inhibit the accumulation of fat in obese individuals.

In addition to these factors, the amount of insulin was measured in HFD mice and mice given 7-MEGA™ since it is well known that an increase in insulin inhibits the burning of fat for energy and promotes the storage of dietary food, primarily as fat. Given their high rates of fat storage, in HFD mice, the results showed that the serum insulin levels were elevated (Fig. 4A). However, 7-MEGA™ lowers insulin levels, suggesting that 7MEGA™ can prevent the accumulation of fat.

### 7‑MEGA™ regulates the adipogenic transcriptional regulators and enzymes in HFD mice

**Fig. 5:**
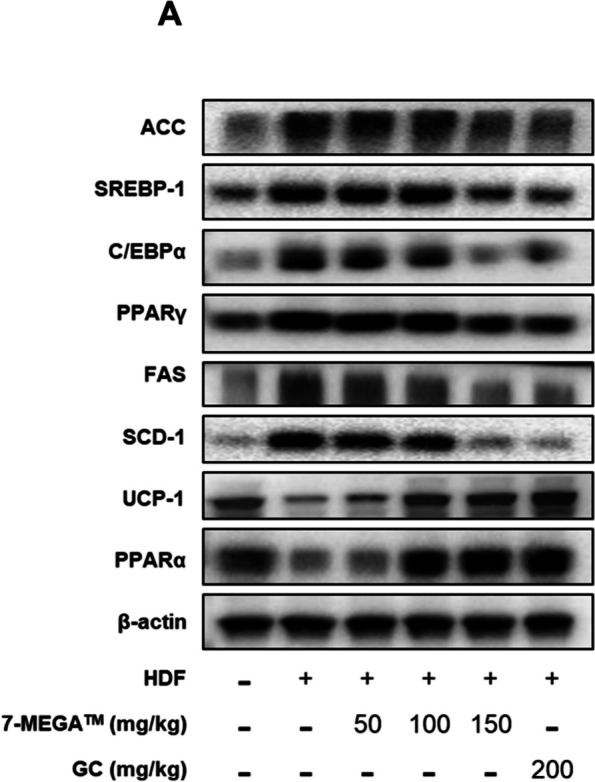
Effects of 7-MEGA™ on adipogenesis-related markers ex vivo. **A** Western blot analysis of protein expression levels of adipogenesis-related markers, PPARγ, C/EBPα, SREBP-1, PPARα, FAS, ACC, UCP-1 and SCD-1 in PWAT of the mice from NC, HFD, and HFD groups orally administered with 50,100 and 150 mg/kg of 7-MEGA™

Finally, the expression of genes that play important roles in obesity was investigated to a more comprehensive understanding of the molecular mechanisms by which 7-MEGA™ is likely to exert inhibitory effects on fat accumulation, adipogenesis, and obesity. PPARγ, PPARα, C/EBPα, SREBP-1, UCP-1, FAS, ACC, and SCD-1 expressions were assessed by western blotting in order to examine the possible suppression of adipogenic transcriptional regulators and enzymes by 7‑MEGA™ in HFD mice. As shown in Fig. 5A, the findings revealed that the protein levels of SCD-1, FAS, C/EBPα, PPARγ, SREBP-1, and ACC reduced when 100 or 150 mg/kg of 7-MEGA™ was administered in HFD groups, indicating the reversal of adipogenic processes. On the other hand, after receiving 100 or 150 mg/kg of 7-MEGA™, the levels of PPARα and UCP-1 increased in HFD groups, suggesting that lipid metabolism had improved in HFD mice thereby ameliorating obesity. These findings demonstrated the potential of 7-MEGA™ to modify lipid metabolism, enhancing the metabolic health of obese individuals and inhibiting obesity.

## Discussion

Obesity presents a significant global health challenge, prompting the exploration of diverse treatment options [32]. According to previous studies, palmitoleic acid (PA) exhibits diverse metabolic benefits in 3T3-L1 adipocytes and obese mice, including increased glucose uptake, enhanced lipolysis via PPARα activation, and anti-inflammatory effects. PA supplementation also improves white adipocyte function, boosts fatty acid oxidation, and mitigates hepatic steatosis in obese mice [16, 18–20]. Thus, the current study, was aimed to investigate the potential therapeutic effects of 7-MEGA™, a purified form of omega-7 containing over 98.5% purity and more than 500 mg/g of PA [25], on both HFD mice and 3T3-L1 adipocytes. 7-MEGATM comprises various fatty acid esters beyond PA (Supplementary Table 1). Individual effects of these esters on obesity have been elucidated in previous studies. For instance, oleic acid has been shown to ameliorate heart conditions by reducing cholesterol levels and inflammation [33]. Additionally, Myristoleic acid has been found to mitigate obesity by activating brown adipose tissue [34]. However, it is important to note that both palmitic acid and myristic acid, as individuals, exhibit adverse metabolic effects that contribute to the development and progression of obesity. These effects can vary depending on factors such as overall dietary composition, metabolic health, and genetic predisposition [35, 36].

Thus, the main objective of this study was to evaluate how 7-MEGA™, as a compound, affects the biology of adipocytes and whether it can help to mitigate the pathophysiology associated with obesity, to evaluate its potential as a functional food, offering health benefits beyond basic nutrition. According to the results from WST-1 assay, 7-MEGA™ demonstrates no significant detrimental impact on adipocyte viability, implying a favorable safety profile in this cellular context. Furthermore, the results of present study showed that 7-MEGA™ treatment inhibits pre-adipocyte differentiation into mature adipocytes in a concentration-dependent manner, suggesting that it may be used as an adipogenesis modulator. This attenuation of adipocyte differentiation was accompanied by a notable reduction in lipid accumulation within the cells, indicative of the efficacy of 7-MEGA™ in mitigating adipocyte hypertrophy and lipid deposition. These findings highlight the intriguing role of 7-MEGA™ in regulating the biology of adipocytes and suggest its potential use as a therapy to treat metabolic dysregulation linked to obesity.

Findings of this study provide more insight into the molecular mechanisms behind the impacts of 7-MEGA™ on the biology of adipocytes and the regulation of lipid metabolism of HFD mice. In particular, the results indicated that higher levels of expression of several key lipogenic and adipogenic markers, such as C/EBPα, PPARγ, SREBP-1, FAS, SCD-1 and ACC in HFD mice and in untreated adipocytes. Since PPARγ is crucial for both lipid metabolism [37] and adipocyte differentiation [38], C/EBPα regulates adipocyte differentiation [39], SREBP-1 is thought to be a transcription factor involved in lipid synthesis [40], FAS contributes to de novo lipogenesis [41], ACC is an essential enzyme in fatty acid synthesis [42], and SCD-1, an enzyme which transforms saturated fatty acids into monounsaturated fatty acids, affecting lipid composition, all of these markers are important for regulating adipogenesis [43]. Following treatment or oral administration of 7-MEGA™, the expression levels of these markers were dramatically lowered, indicating that the supplement may have the ability to inhibit adipogenesis. Furthermore, the expression levels of FABP4, which is primarily expressed in adipocytes, increased in adipocytes and decreased upon 7-MEGA™ treatment implying the potential of 7-MEGA™ to inhibit adipogenesis.

Moreover, PPARα and UCP-1 expressions were at lower levels in obese mice in this investigation. However, the administration of 7-MEGA™ increased their expression. PPARα promotes lipid metabolism and helps to avoid adipogenesis by controlling gene expressions involved in fatty acid oxidation [44]. UCP-1 serves a critical function in thermogenesis, promoting energy expenditure and preventing obesity [45]. The administration of 7-MEGA™ restored their expression indicating that 7-MEGA™ promote energy expenditure and lipid oxidation. These findings imply that 7-mega may improve fatty acid oxidation and thermogenesis to prevent obesity-induced metabolic dysregulation, helping to treat obesity and its related metabolic problems.

Furthermore, according to the results, in HFD mice, 7-MEGA™ treatment led to a concentration-dependent decrease in body weight and food consumption. The observed reduction in food intake among obese mice may be attributed to its potential anti-obesity effects, potentially mediated through mechanisms such as appetite suppression, alteration of metabolic pathways, or modulation of hormonal signalling related to hunger and satiety. All of these results suggest the possibility that 7-MEGA™ might successfully prevent obesity-related weight gain and adiposity by reducing fat formation, decreasing appetite, and possibly preventing the differentiation of adipocytes.

Findings further demonstrated that the HFD group had larger adipocytes, and size was normalized by the administration of 7-MEGA™. The primary cause of this increase in adipocyte size is a condition known as adipocyte hypertrophy, which is resulting from an overabundance of accumulation of triglycerides and other lipids within the adipocytes [30]. Additionally, it was noted that the treatment of 7-MEGA™ caused the steatosis in the liver tissues caused by the accumulation of fat in the livers of obese mice disappeared. Obesity and steatosis are frequently linked [46, 47]. The findings further demonstrated that 7-MEGA™ can lessen the accumulation of fat mass in the adipose tissues of PWAT and SWAT. All of these concentration-dependent effects reflect the possible dose-dependent effectiveness of 7-MEGA™ in the treatment of metabolic disorders associated with fat accumulation and obesity.

In the HFD group, lower levels of adiponectin and higher levels of insulin, leptin, IFN‑γ, TNF‑α, IL‑6 and IL‑1β were observed. On the other hand, the administration of 7-MEGA™ caused these biomarkers to shift, leading to a rise in adiponectin levels and a drop-in insulin, leptin, IFN‑γ, TNF‑α, IL‑6 and IL‑1β. These changes indicate a beneficial shift in the metabolic environment, marked by an increase in anti-inflammatory adiponectin and a decrease in pro-inflammatory cytokines and insulin resistance indicators. More specifically, increased adiponectin indicates greater insulin sensitivity and maybe improved metabolic health, while decreased insulin, leptin, IFN‑γ, TNF‑α, IL‑6 and IL‑1β levels suggest attenuation of inflammation and improved insulin sensitivity [48, 49]. All of these modifications suggest that 7-MEGA™ may be able to reduce metabolic dysregulation linked to obesity by influencing inflammatory pathways and adipokine levels.

As a summary, the aim of this study was to investigate the therapeutic potential of 7-MEGA™ in the management of obesity and related metabolic disorders. The results showed that in HFD mice, 7-MEGA™ administration reduced body weight and food intake along with decreasing the number of primary adipocytes and fat mass accumulation. Additionally, 7-MEGA™ administration resulted in the modulation of key adipogenic and lipogenic markers, including reduced expression levels of PPARγ, FAS, ACC, C/EBPα, SREBP1, and SCD-1, exploring its capacity to mitigate adipocyte differentiation and lipogenesis. Furthermore, 7-MEGA™ treatment reversed the downregulation of thermogenic and fatty acid oxidation indicators, such as UCP-1 and PPARα, and mitigated the dysregulation of adipocyte-specific markers, such as FABP4. This suggests that 7-MEGA™ has a role in increasing energy expenditure and lipid oxidation. In addition, the 7-MEGA™ treatment caused alterations in circulating biomarkers, which showed an elevation in adiponectin levels and a decrease in insulin, leptin, IFN‑γ, TNF‑α, IL‑6 and IL‑1β. These changes suggested an overall improvement in metabolic health and decrease in inflammation. All things considered, this research emphasises the complex ways that 7-MEGA™ affects the biology of adipocytes and the control of metabolism. To completely clarify the therapeutic efficacy of 7-MEGA™ in clinical settings, more research into the molecular processes underlying these reported results is necessary.

Furthermore, the understanding the impact of 7-MEGA™ on adipogenesis in animals on high-fat diets involves considering its potential benefits and risks within the complex framework of metabolic regulation. While the ability of 7-MEGA™ to inhibit adipogenesis offers promise in reducing fat accumulation, especially pertinent in addressing obesity, it is essential to acknowledge the intricate role of adipose tissue in metabolic homeostasis. Adipogenesis is critical for energy balance, lipid metabolism, and hormonal regulation [50]. Disrupting this process entirely could lead to metabolic dysregulation and associated health risks, particularly in individuals already susceptible to metabolic disturbances [51, 52]. Therefore, the use of 7-MEGA™ or similar compounds in animals on high-fat diets demands careful evaluation of both its potential advantages and the broader implications for metabolic health. Further research is necessary to comprehensively understand the effects of inhibiting adipogenesis in these contexts, guiding more informed therapeutic strategies to improve metabolic health in affected populations.

### Strengths and limitations of the study

This study benefits from its comprehensive approach to investigating the therapeutic potential of 7-MEGA™ in managing obesity and related metabolic disorders. The research employs a combination of in vivo and in vitro experiments, providing a multifaceted understanding of how 7-MEGA™ influences adipocyte biology and metabolic processes. Additionally, the study utilizes a purified form of omega-7 with high purity, enhancing the reliability and consistency of the results. Moreover, the investigation explores various molecular markers and circulating biomarkers, offering insights into the mechanisms underlying the observed effects of 7-MEGA™. Despite its strengths, this study has several limitations that should be considered. Firstly, the research primarily focuses on animal models and adipocyte cultures, limiting the direct applicability of the findings to human populations. Furthermore, the study lacks long-term follow-up data, making it challenging to assess the sustained efficacy and safety of 7-MEGA™ over extended periods.

## Conclusions

The findings of this study hold significant clinical relevance for managing obesity and related metabolic disorders, suggesting that 7-MEGAT^M^ may serve as a valuable therapeutic agent. By reducing adipogenesis, lipid accumulation, and inflammatory biomarkers, 7-MEGA™ shows promise in improving metabolic health and addressing obesity-related complications. Additionally, understanding its molecular mechanisms offers insights for targeted interventions. In clinical practice, 7-MEGA™ could be utilized as a functional food ingredient to promote weight loss and enhance metabolic health. Future research should focus on further elucidating its efficacy and safety in diverse populations through clinical trials. Overall, 7-MEGA™ has the potential to be a key component in functional foods aimed at combating obesity and metabolic syndrome.

### Supplementary Information

Supplementary Material 1: Supplementary Table 1. Typical fatty acid profiles of 7‑MEGA™.

## Data Availability

No datasets were generated or analysed during the current study.

## Abbreviations

ACC: Acetyl-coA carboxylase
BAT: Brown adipose tissue
C/EBPα: CCAAT/enhancer-binding protein alpha
COX2: Cyclooxygenase-2
FABP4: Fatty acid binding protein 4
H2O2: Hydrogen peroxide
HFD: High-fat diet
IL: Interleukin
MMP3: Matrix Metalloproteinase 3
PWAT: Parametrial white adipose tissue
SCD-1: Stearoyl-CoA Desaturase-1
SWAT: Subcutaneous white adipose tissue
UCP-1B: Uncoupling Protein 1
WAT: White adipose tissues
