# Supplementary material for: 7-MEGA™ inhibits adipogenesis in 3T3-L1 adipocytes and suppresses obesity in high-fat-diet-induced obese C57BL/6 mice
Source: Lipids Health Dis. 2024 Jun 22;23:192. doi: 10.1186/s12944-024-02175-0 (PMC11193219; doi:10.1186/s12944-024-02175-0)
Supplement: Supplementary file 1 — Supplementary Material 1: Supplementary Table 1. Typical fatty acid profiles of 7‑MEGA™. [file 12944_2024_2175_MOESM1_ESM.docx]

**Supplementary table 1:** Typical fatty acid profiles of 7‑MEGA™

| **Fatty acid ethyl esters - Common name** | **(% w/w)** |
| --- | --- |
| Palmitoleic | 50.52 |
| Palmitic | 20.35 |
| Unidentified C 16 & Cl 8 polyunsaturated ethyl esters | 16.62 |
| Oleic | 5.31 |
| Myristoleic | 0.48 |
| Myristic | 0.08 |
| Myristelaidic | 0.02 |
| Stearic | 0.11 |
| trans-octadecenoic | 0.93 |
| cis-Vaccenic | 2.01 |
| Linoleic | 0.56 |
| Gamma-linolenic | 0.06 |
| Alpha-linolenic | 0.31 |
| Stearidonic | 0.77 |
| Gadoleic | 0.04 |
| Gondoic | 0.02 |
| EPA | 0.11 |
| Docosanoic | 0.04 |
| Docosapentaenoic | 0.03 |
| DHA | 0.20 |
